# Robot-assisted jaw-in-a-day based on the sandwich approach: case series

**DOI:** 10.1186/s40902-026-00513-z

**Published:** 2026-05-28

**Authors:** Ping Luo, Xiang Gao, Lingyu Su, Hongying Chen, Jiang Deng, Xiaochun Ren, Gang Fu, Yuanding Huang, Zhanpeng Ou, Fugui Zhang

**Affiliations:** 1 Department of Oral and Maxillofacial Surgery, First Affiliated Hospital of Chongqing Medical University, Chongqing, China; 2 Department of Oral and Maxillofacial Surgery, Affiliated Stomatological Hospital of Chongqing Medical University, Chongqing, China; 3 Chongqing Key Laboratory of Oral Diseases, Chongqing, China; 4 Chongqing Municipal Health Commission Key Laboratory of Oral Biomedical Engineering, Chongqing, China

**Keywords:** Robot-assisted surgery, Jaw-in-a-day, Mandibular reconstruction, Dental implant, Sandwich approach, Fibula flap, Iliac flap

## Abstract

**Background:**

The “jaw-in-a-day” (JIAD) concept involves integrating the resection of mandibular tumors with mandibular defect repair and immediate implant-supported prosthetic rehabilitation into one surgical operation. This approach aims to reduce the overall treatment cycle, with the goal of improving patients’ quality of life. However, traditional JIAD frequently employs static surgical guides, which can introduce variability in implant placement accuracy. Robot-assisted surgery has emerged as a promising alternative, with the potential to improve surgical precision through real-time navigation and haptic feedback technology.

**Case presentation:**

This retrospective case series reported five cases of mandibular tumors treated using the robot-assisted JIAD. Surgical planning involved virtual surgical simulation and computer-aided design/manufacturing based on sandwich planning technology. A robotic surgical system was utilized to prepare the implant site and place the implant under real-time navigation guidance. Immediate provisional prostheses were placed during the same surgery. All robot-assisted JIAD surgeries were performed as planned. The robot-assisted preparation and placement of implant sites yielded a mean angular deviation of 1.17° ± 0.30° and a linear deviation of less than 0.5 mm. Radiographic analysis at six months post-surgery revealed signs consistent with implant stability and osseointegration.

**Conclusion:**

The robot-assisted JIAD technique, based on the sandwich planning approach, appears to be a feasible approach that may enhance implant placement accuracy in complex mandibular reconstructions. However, these preliminary findings require further validation in larger, controlled studies.

**Supplementary Information:**

The online version contains supplementary material available at 10.1186/s40902-026-00513-z.

## Introduction

Mandibular defects commonly arise from tumor resection, trauma, osteomyelitis, osteoradionecrosis (ORN), or medication-related osteonecrosis of the jaw (MRONJ) [1]. Reconstruction aims not only to restore skeletal continuity but also to facilitate the recovery of vital functions such as mastication, swallowing, and speech, while promoting favorable facial aesthetics [2]. Vascularized free flaps, particularly fibular flaps and iliac flaps, are considered the gold standard for such reconstructive procedures [1, 3]. Nevertheless, traditional restoration approaches typically involve multiple surgeries over an extended period. This predictably leads to extended edentulism, as well as masticatory, aesthetic, and quality of life impairments and increases cumulative surgical risks and healthcare expenditures [4, 5].

Over the last few years, the notion of jaw-in-a-day (JIAD), facilitated by virtual surgical planning (VSP), computer-aided design/computer-aided manufacturing (CAD/CAM) technology, and provision of a fixed temporary prosthesis, has allowed for the integration of tumor ablation, microvascular bone flap repair, dental implant placement, and delivering a fixed prosthesis to be completed in a single surgery, thereby reducing the overall length of the treatment period [6, 7]. Its core advantage lies in minimizing the edentulism period and facilitating early functional recovery after surgery, thereby potentially benefiting patients’ quality of life [8, 9].

Nevertheless, traditional JIAD surgical protocols rely heavily on conventional static surgical guides in the case of implant site preparation and implant placement. The precision of this technique depends on various aspects, including the design of the guide plate, guide plate positioning, manual stability, the fit between the titanium plate and bone surface, soft tissue interference, and surgical experience, all of which can result in deviations in the angle, depth, and three-dimensional (3D) orientation of the implant. Such inaccuracies may have a negative impact on the passive fit of the prostheses as well as the long-term stability of the implants [6, 10]. In addition, the fibular flap, widely used in JIAD surgery, has inherent limitations in bone height and width, which greatly increases the complexity of implant planning. Improper handling may lead to insufficient restorative space or even implant failure [11, 12]. To overcome these constraints, digital workflows have evolved towards more dynamic and automated solutions. For instance, the integration of intraoral surgical navigation systems with specialized techniques, such as piezo surgery, holds promise in supporting 3D implant positioning [13].

Robot-assisted surgery (RAS) has showed its innovative technological value in the surgical field through its exceptional precision, operational stability, and highly repeatable procedures [14, 15]. Robotic systems are utilized in oral implantology to provide an uninterrupted flow between the 3D preoperative planning and intraoperative real-time navigation, haptic feedback, and scale of motions. This integration allows using preplanned implant placements with an accuracy of less than one millimeter, even in anatomically complex conditions [16]. Preliminary research indicates that the application of RAS in jaw reconstruction holds significant potential for enhancing the precision of osteotomy procedures and restoring facial contours [17]. Pioneering studies have also shown that, compared to computer-assisted navigation techniques and traditional free-hand techniques, robot-assisted mandibular reconstruction using a fibular flap offers greater precision in bone segment positioning and osteotomy [18]. However, the application of RAS in the complex workflow of JIAD surgery, particularly for optimizing implant positioning and prosthesis placement during free flap procedures, has limited documentation.

This study aims to investigate the feasibility of combining robot-assisted technology with the sandwich design concept in JIAD and to measure the actual precision of the implants.

## Case presentation

### Patient demographics

This case series included five patients with mandibular tumors who underwent robot-assisted JIAD reconstruction for segmental mandibular defects, between March 2024 and April 2025 (Table 1). The study protocol was approved by the Institutional Review Board (2023 (198)) and conducted in accordance with the Declaration of Helsinki. Written Informed Consent was obtained from all patients following a comprehensive preoperative evaluation.

**Table 1:** Patient demographics and surgical information

Case No.	Sex	Age (years)	Pathology	Flap Type	Number of Implants	Implant Positions	Restored Teeth
1	F	36	OF	Fibula	2	36, 37	36–37
2	F	54	OM	Fibula	4	43, 41, 35, 36	43 − 36
3	F	51	CCOC	Iliac	3	44, 45, 47	43–47
4	F	43	AM	Iliac	4	34, 36, 41, 43	43 − 36
5	M	38	GCG	Iliac	4	44, 45, 46, 47	43–47

*Abbreviations: M* Male, *F* Female, *CCOC* Clear cell odontogenic carcinoma, *OF* Ossifying fibroma, *AM* Ameloblastoma, *OM* Odontogenic myxofibroma, *GCG* Giant cell granuloma

### VSP

All patients underwent preoperative contrast-enhanced computed tomography (CT) scans and cone-beam CT (CBCT) of the head and neck, as well as contrast-enhanced CT scans of the respective donor sites (fibula or iliac bone). VSP was performed using dedicated software (ProPlan CMF 3.0, Materialise, Belgium) to simulate tumor resection. Following the identification of the tumor’s location and extent, the mandibular osteotomy guides were designed. These cases involved the use of the sandwich approach. It is defined as a technique that engages planning the positions of grafted bone and the prosthesis before planning the positions of dental implants. According to the size and shape of the defect, iliac flaps (3 cases) or fibular flaps (2 cases) were selected for mandibular defect reconstruction. The unaffected contralateral mandible was reflected, and it was used as a directive in the reconstruction of defects. The bone fragments that had been reconstructed were placed approximately 12–15 mm below the occlusal plane to provide sufficient restorative dimensionality, and osteotomy guides were then designed. Based on the concept of occlusion-driven design as previously described [19], the optimal implant type, position, angle, and insertion depth were determined using implant planning software (3Shape Implant Studio, 3Shape, Denmark). The final virtual plan, which included the reconstructed mandible and implant positions, was exported and then imported into the robotic surgery system control software (Remebot, China). The trajectory of the robotic arm and the implantation sequence into the system were previously simulated to eliminate the risk of collision of instruments and avoid interference with critical anatomical structures. Based on the definitive VSP plan, stereolithography technology was used to 3D print models of the deficient mandible, the reconstructed mandible, and surgical guides for mandibular and graft bone osteotomy and reconstruction.

### Surgical procedure

All the procedures were carried out under general anesthesia. The surgeries commenced with the resection of the mandibular tumors, guided by a preoperatively designed mandibular osteotomy plate. The preparation of the mandibular defect was done with exposing the bone ends, dissection, and protecting adjacent recipient vessels. When simultaneously harvesting a vascularized iliac or fibular flap, it was necessary to carefully preserve its vascular pedicle as previously described [20]. The shaped flap was placed into a 3D-printed mandibular defect model, and the bone flap was fixed to the model with mini-titanium plates. Subsequently, registration was performed using preset positioning markers, and the robotic system was calibrated. After completing registration and calibration, the robotic system was utilized to guide implant site preparation and placement procedures under real-time navigation and haptic feedback, in accordance with the preoperative plan. This facilitated a close alignment of the 3D position, angle, and depth of each implant with the virtual design. Following robot-assisted implant placement, a prefabricated 3D-printed positioning template was used to verify the passive fit and spatial orientation of the temporary prosthesis. This process formed an integrated “bone flap – implant – temporary denture” complex. Subsequently, this complex was transplanted to the mandibular defect site. Microvascular anastomosis was performed under the surgical microscope to restore the blood supply to the flap. The bone flap was firmly fixed to the defected mandible using pre-bent mini-titanium plates.

Ultimately, fixed temporary dentures were installed. In this case series, a non-functional immediate restoration approach was employed. Although fixed temporary prostheses were placed during the same surgical procedure, they were designed to remain in a non-occlusal position. Meticulous intraoperative occlusal adjustments were performed to eliminate any premature contact with the opposing dentition. This approach is intended to protect vascularized bone grafts from excessive masticatory forces during the early stages of bone healing and osseointegration.

Postoperatively, all patients underwent routine intermaxillary elastic traction for approximately four weeks. This measure was consistently applied across the cohort to stabilize the intermaxillary relationship, maintain the planned occlusion, and provide an optimal environment for the consolidation of the reconstructed segments.

### Robot-assisted JIAD cases utilizing fibula flap reconstruction

**Fig. 1:**
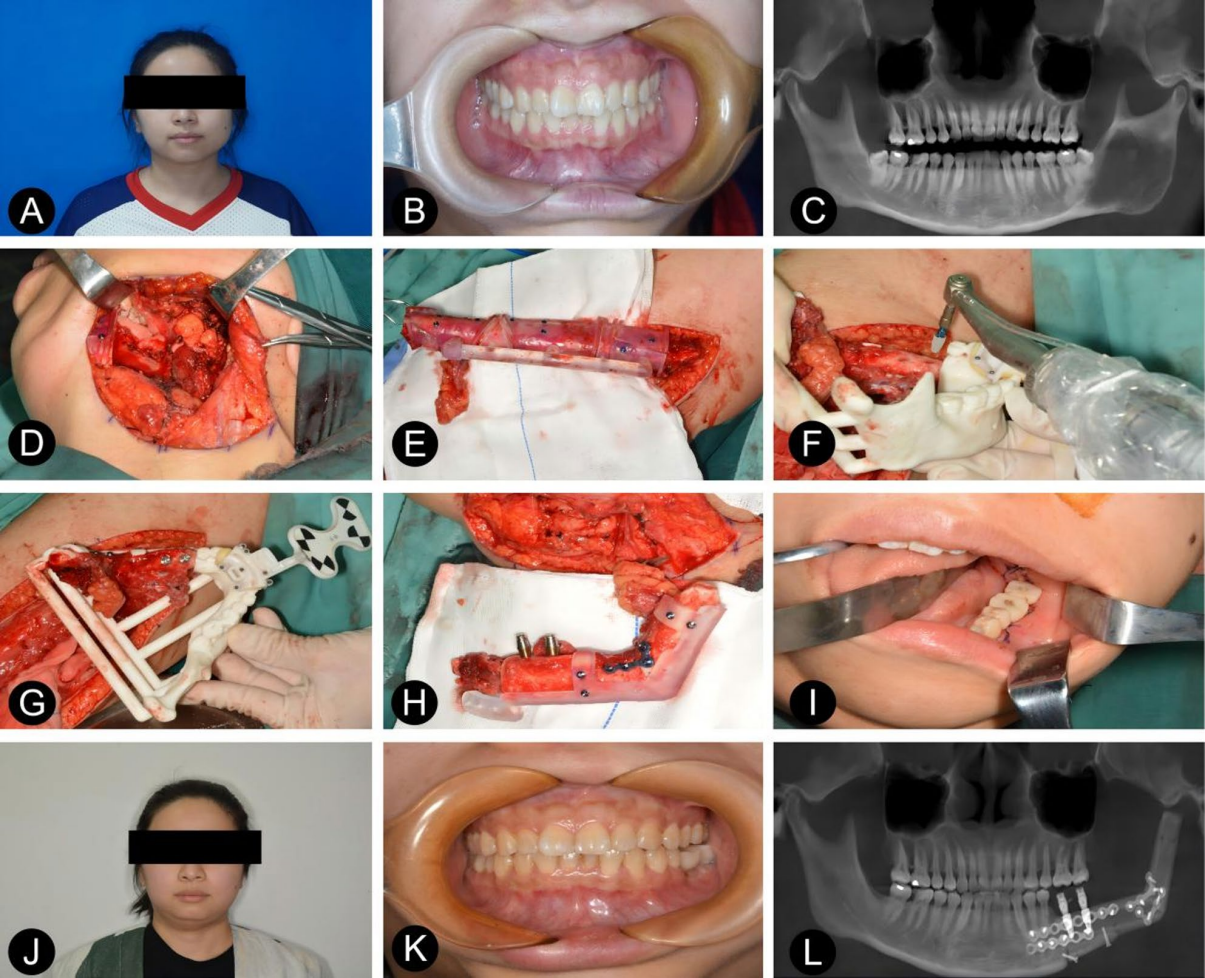
Case 1 of robot-assisted fibular flap JIAD. **A**–**C**: Preoperative appearance, intraoral occlusion, and preoperative panoramic film. **D**–**I**: Intraoperative guide-assisted resection of mandibular lesions and harvest of a fibula flap. Robot-assisted simultaneous implant placement in a mandibular defect model with the help of a fiducial marker and fixed denture restoration of the dentition. **J**–**L**: Postoperative 6-month follow-up examination of facial appearance, intraoral occlusion, and postoperative imaging

Case 1: A 36-year-old female patient was diagnosed with an ossifying fibroma in the left mandible through radiographic imaging and histopathological biopsy. Following multidisciplinary team discussion, a robot-assisted JIAD procedure was planned. The mandibular defect involved the left mandibular body and the entire left ramus, which was reconstructed with a double-barrel fibula flap. Two implants (teeth 36 and 37) were placed simultaneously, followed by immediate provisional prosthesis restoration. Both implants and the prosthesis remained functional at the 3- and 6-month follow-up evaluations (Fig. 1).

**Fig. 2:**
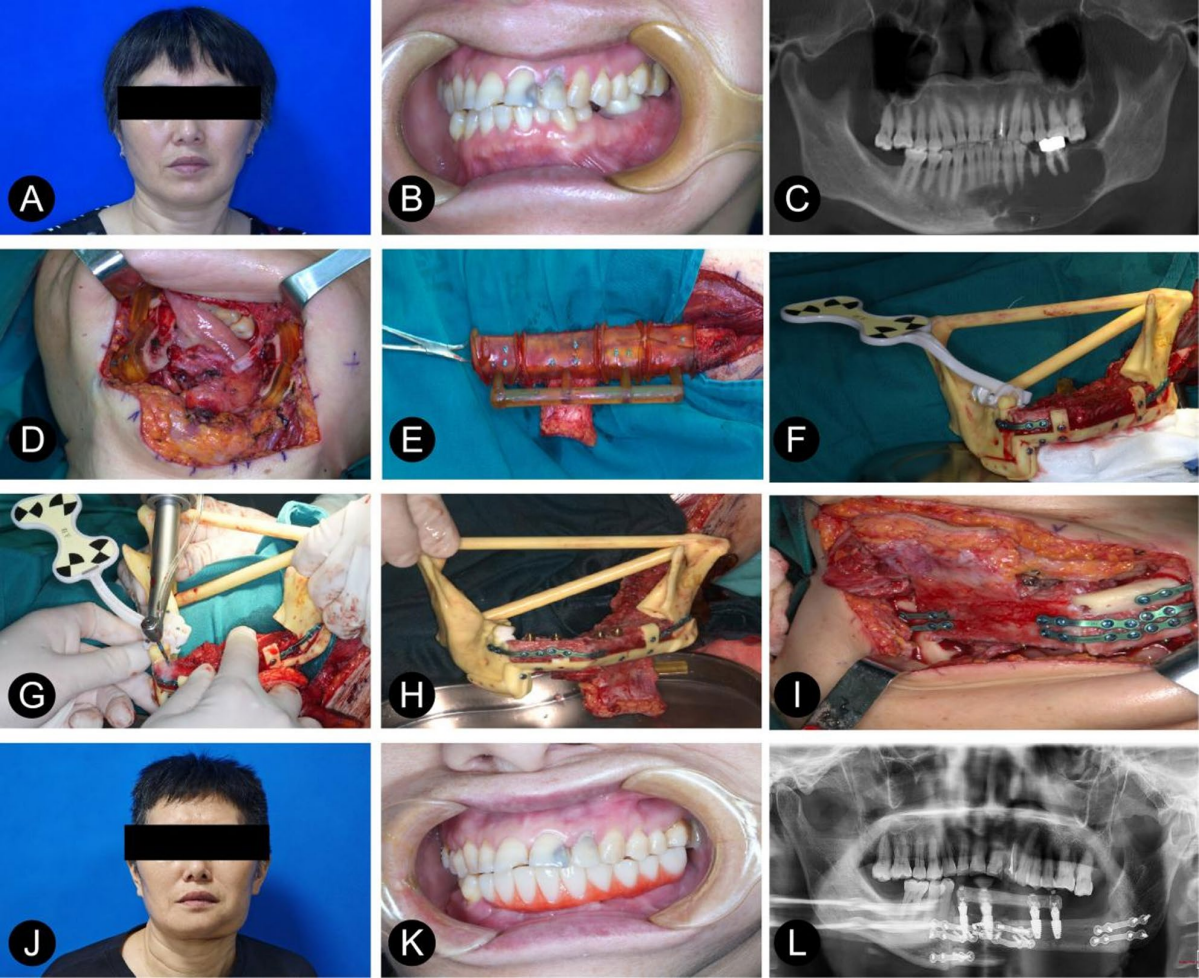
Case 2 of robot-assisted fibular flap JIAD in a patient with odontogenic myxofibroma. **A**–**C**: Preoperative appearance, intraoral occlusion, and preoperative imaging. **D**–**I**: Intraoperative guide-assisted resection of mandibular lesions and preparation of fibula bone grafts and robot-assisted simultaneous implant site preparation and placement. **J**–**L**: Postoperative 2-month follow-up examination of facial appearance, intraoral occlusion, and postoperative 6-month imaging

Case 2: A 54-year-old female patient presented with swelling and discomfort in the left mandible. The pathological diagnosis was odontogenic myxofibroma. The mandibular defect ranged from tooth 43 to 37. A two-segment double-barrel fibula flap was employed for JIAD reconstruction, with four implants placed concurrently. A fixed provisional prosthesis was delivered postoperative week three (Fig. 2).

### Robot-assisted JIAD cases with iliac flap reconstruction

**Fig. 3:**
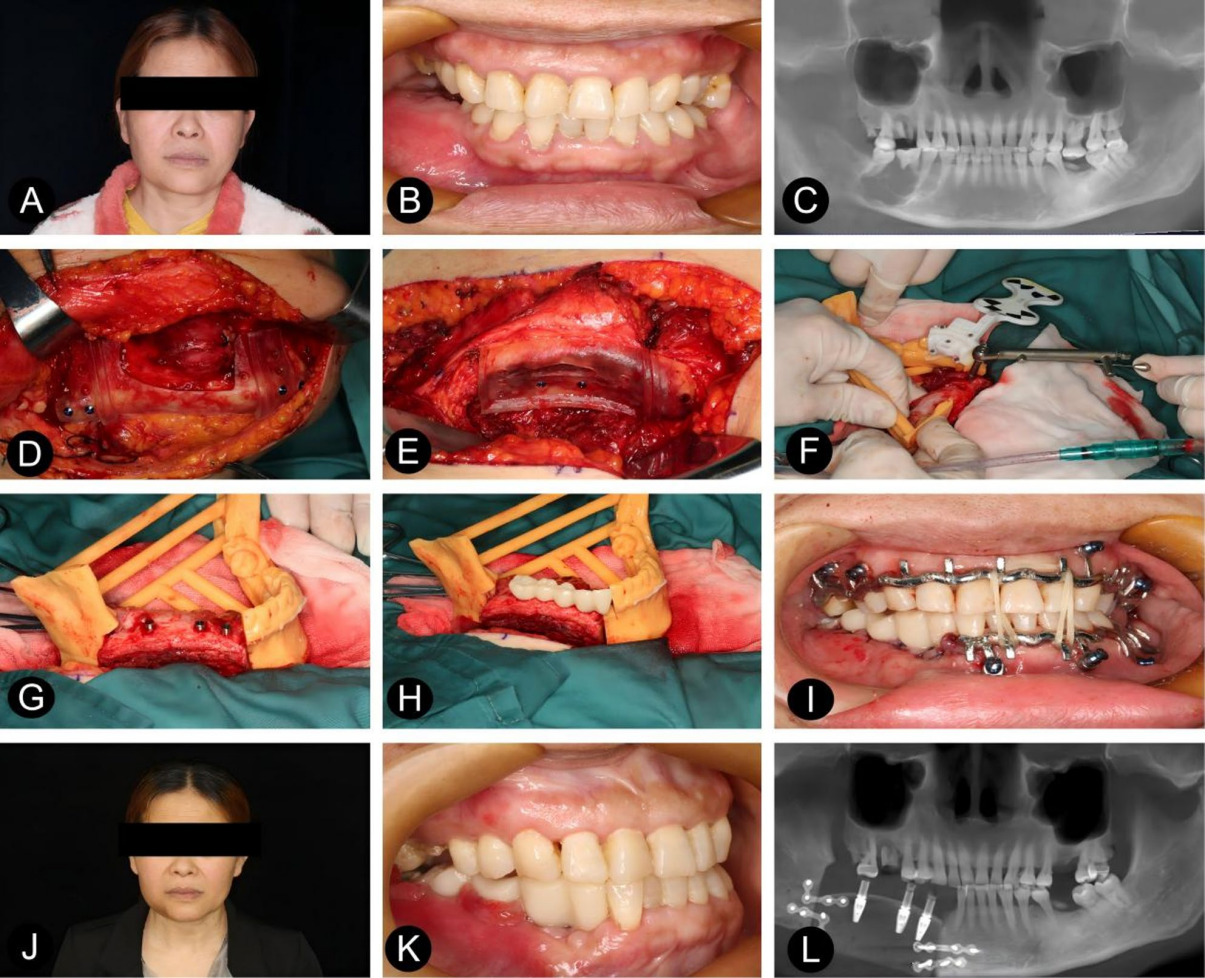
Case 3 of robot-assisted iliac flap JIAD. **A**–**C**: Preoperative appearance, intraoral occlusion, and preoperative imaging. **D**–**I**: Intraoperative guide-assisted mandibular lesion resection and harvest of an iliac bone flap. Robot-assisted simultaneous implant placement, fixed denture restoration, and intermaxillary fixation with a pair of personalized dental arch bars. **J**–**L**: Postoperative 6-month follow-up examination of facial appearance, intraoral occlusion, and postoperative imaging

Case 3: A 51-year-old female patient presented with a clear cell odontogenic carcinoma (preoperative biopsy indicated an ameloblastoma) in the right posterior mandible, diagnosed via CBCT and biopsy. The defect spanned the mandibular body corresponding to teeth 43 to 47. A single-segment iliac flap was utilized for JIAD reconstruction. Three implants were placed simultaneously, followed by immediate fixed provisional restoration. Both implants and the prosthesis remained functional throughout the 3- and 6-month follow-up assessments. No recurrence of the tumor was found during follow-up (Fig. 3). In addition, because the postoperative pathological diagnosis was malignant tumor (CCOC), the patient underwent more intensive oncological monitoring; during the 22-month follow-up period, no signs of tumor recurrence were observed.

**Fig. 4:**
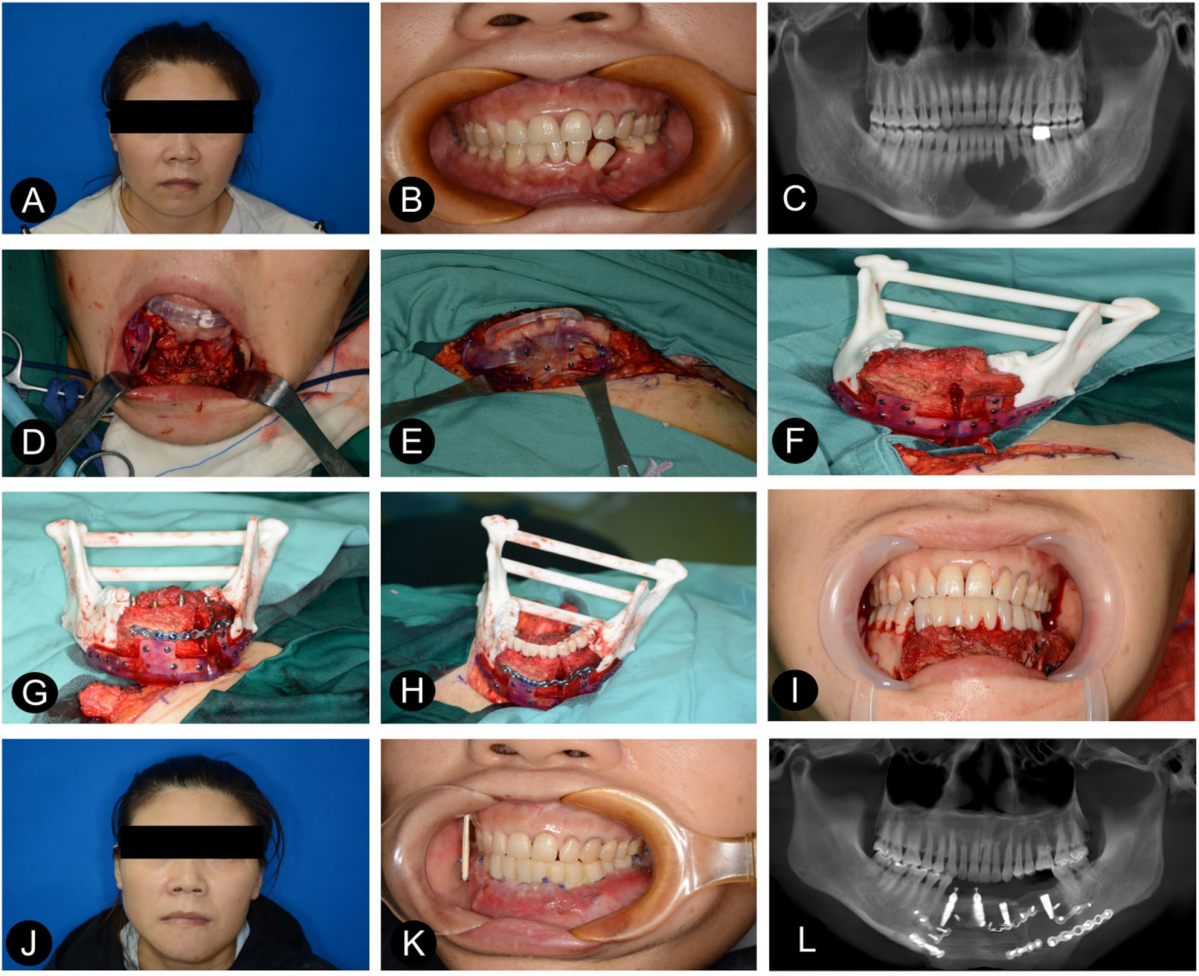
Case 4 of robot-assisted iliac flap JIAD in a patient with ameloblastoma. **A**–**C**: Preoperative appearance, intraoral occlusion, and preoperative imaging. **D**–**I**: Intraoperative guide-assisted mandibular lesion resection and iliac bone flap preparation. Robot-assisted simultaneous implant placement and extraoral and intraoral fixed denture restoration. **J**–**L**: Postoperative 1-month follow-up examination of facial appearance, intraoral occlusion, and postoperative 6-month imaging

Case 4: A 43-year-old female patient was admitted for mandibular ameloblastoma. During surgery, the mandibular segment corresponding to teeth 43 to 36 was resected. A two-segment iliac flap was used to reconstruct the mandibular defect. Four implants were placed simultaneously. At 6-month postoperative follow-up, the implants were stable with favorable occlusal relationships (Fig. 4).

**Fig. 5:**
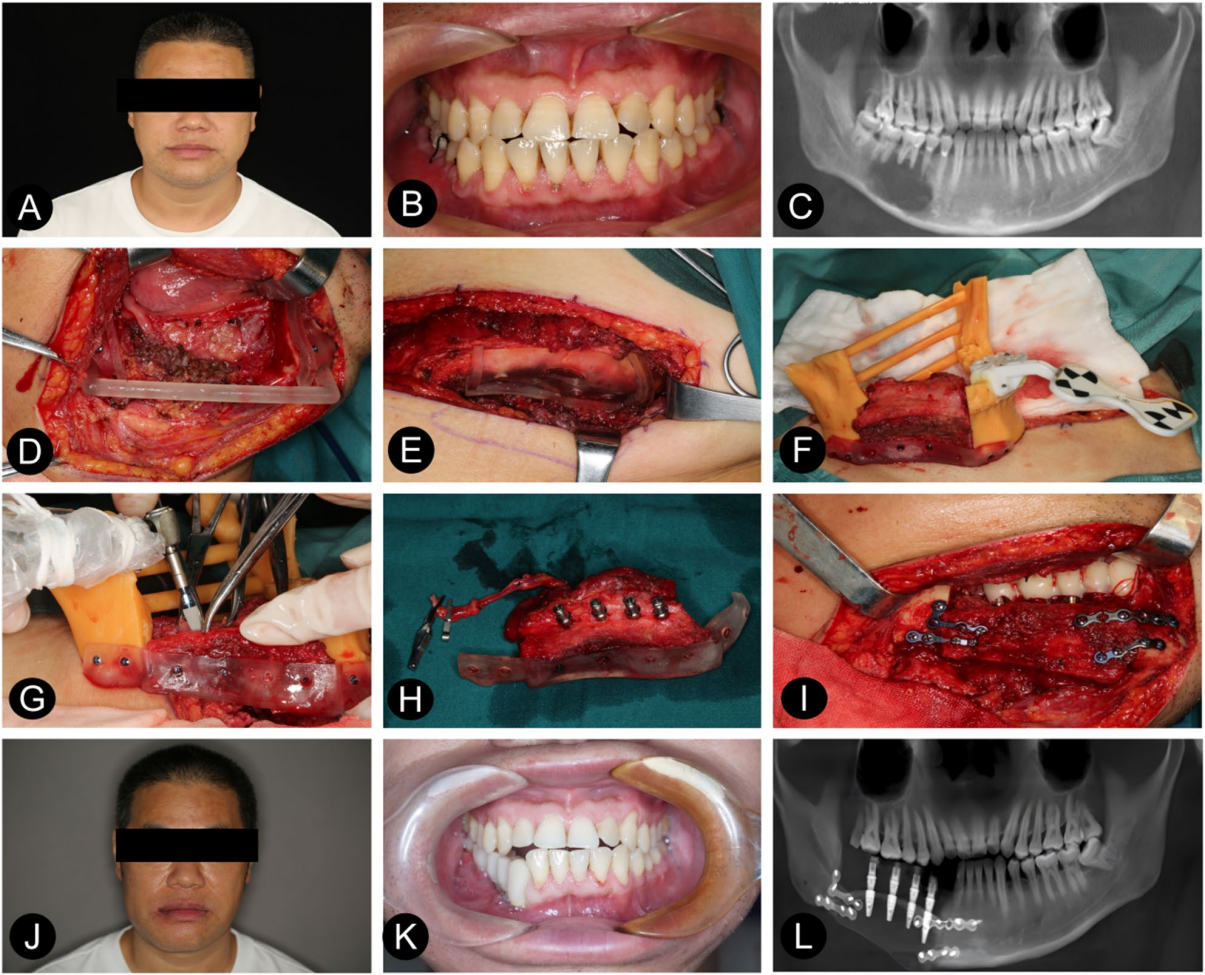
Case 5 of robot-assisted iliac flap JIAD in a patient with giant cell granuloma. **A**–**C**: Preoperative appearance, intraoral occlusion, and preoperative imaging. **D**–**I**: Intraoperative guide-assisted mandibular lesion resection and iliac bone flap preparation. Robot-assisted simultaneous implant placement and fixed denture restoration. **J**–**L**: A 3-month postoperative follow-up examination of facial appearance, intraoral occlusion, and postoperative 6-month imaging

Case 5: A 38-year-old male patient was admitted for giant cell granuloma. During surgery, a segment of the mandible corresponding to teeth 43 to 47 was ablated. A single-segment iliac flap was used to reconstruct the mandibular defect, and four dental implants were simultaneously placed. At 6-month postoperative follow-up, the implants were stable and the occlusion was well-aligned (Fig. 5).

## Results

Quantitative accuracy analysis was conducted by superimposing postoperative CBCT images with preoperative VSP using the robot’s integrated postoperative verification module (Remebot, China). This module is a dedicated software tool designed for objective accuracy validation, it performs a high-precision registration between the preoperative plan and the postoperative DICOM data using a point-cloud matching algorithm. Once the coordinates are aligned, the system automatically calculates the 3D vector deviations at the entry point (implant abutment) and the apical point (implant apex), as well as the overall angular deviation between the planned and actual implant longitudinal axes.

The accuracy analysis was independently performed by two investigators experienced in robotic system software. Although no formal inter- or intra-observer reliability statistics (e.g., Intraclass Correlation Coefficient, ICC) were calculated, each measurement was independently repeated twice by both investigators and averaged to minimize measurement error. While the investigators were not blinded to the surgical plan, the measurement process relies on a semi-automated algorithm within the robotic system, which is designed to minimize subjective manual error and potential observer bias. Furthermore, given the descriptive nature of this retrospective case series, no inferential statistical analyses were performed.

As summarized in Table 2, all 17 implants placed in the five cases showed a mean overall angular deviation of 1.17° ± 0.30°, a mean entry point deviation of 0.26 ± 0.09 mm, and a mean apical deviation of 0.24 ± 0.08 mm. No implant exceeded the predefined safety thresholds of 2° for angular deviation or 0.5 mm for linear deviation.

**Table 2:** Quantitative analysis of implant placement accuracy in robot-assisted JIAD

Case No.	Flap Type	No. of Implants	Angular deviation (°)	Entry point deviation (mm)	Apical deviation (mm)
1	Fibula	2	1.25 ± 0.20	0.28 ± 0.10	0.27 ± 0.09
2	Fibula	4	1.40 ± 0.30	0.32 ± 0.12	0.30 ± 0.11
3	Iliac	3	1.20 ± 0.30	0.25 ± 0.08	0.22 ± 0.07
4	Iliac	4	0.90 ± 0.40	0.19 ± 0.06	0.17 ± 0.05
5	Iliac	4	1.10 ± 0.30	0.24 ± 0.09	0.23 ± 0.08
Total/average	-	17	1.17 ± 0.30	0.26 ± 0.09	0.24 ± 0.08

## Discussion

The traditional JIAD technique, due to technical limitations, is generally considered more appropriate for patients with favorable anatomical structures and sufficient bone volume [8]. In more complex cases involving large defects, proximity to adjacent anatomical structures, or the use of morphologically intricate flaps, conventional guides may present limitations. RAS technology, characterized by three-dimensional spatial perception, obstacle avoidance capabilities, and force feedback control, is designed to facilitate operations in complex anatomical areas with enhanced safety and accuracy [14]. This has facilitated the management of cases previously considered relative contraindications for iliac flap transplantation. In this case series, robot-assisted iliac and fibular flap transplantations were performed for various mandibular defect morphologies, suggesting the potential adaptability of this technique.

The precision of implant site preparation and placement is fundamental to the success of immediate implant supported occlusal restoration [21]. Traditional JIAD, which requires free-hand actions, is also prone to accurate errors due to various factors, such as guide design, fabrication, intraoperative termination, and surgeon experience, where the literature indicates an implant angular deviation of 2°–4° and a linear deviation of 1–2 mm [6, 10, 22]. These deviations present potential clinical challenges, particularly in cases of limited bone stock, as they might compromise the prosthesis or introduce long-term risks. Our robot-assisted series yielded a mean angular deviation of 1.17° ± 0.30° and an apical deviation of 0.24 ± 0.08 mm. Although this study does not include a direct control group, our findings align with the high-precision outcomes expected from robotic systems [10, 21, 23, 24].

JIAD is primarily indicated for benign tumors or borderline tumors (benign tumors with aggressive behavior, such as ameloblastoma), where immediate restoration does not compromise oncological surveillance. Although Case 3 was postoperatively diagnosed as CCOC (a rare malignancy in the mandible), the preoperative biopsy indicated an ameloblastoma; thus, JIAD was initiated based on a benign diagnosis. We do not advocate JIAD for known or suspected malignancies. In case 3, a 1 cm radical margin was intended to be maintained during resection, which appeared to have been effective, as no recurrence occurred during the 22-month follow-up. Nonetheless, this case highlights the risk of diagnostic discrepancy between biopsy and final pathology. If malignancy is unexpectedly found postoperatively, intensive follow-up is of great importance. In our practice, staged reconstruction is generally selected for pathologically diagnosed malignancies to support adequate oncological management.

Although robotic systems are primarily used to address spatial constraints and limited visibility within the oral cavity, our implant placement procedures are performed on an in-vitro workbench. In this unrestricted environment, traditional 3D-printed static guides might provide comparable precision at a lower cost and with simpler operation. Nevertheless, we opted for robot-assisted surgery primarily due to its capacity for real-time adaptability. If adjustments to bone segments, implant positions, or orientations are required during surgery, the surgeon can immediately update the virtual surgical plan, whereas a pre-designed static guide would become ineffective. We acknowledge that, in an ex vivo setting, the added value of robotic assistance over static guides has not yet been demonstrated. Future direct comparative studies are needed to evaluate its potential clinical benefits in this context.

Although the JIAD with the assistance of robots has tremendous benefits, there are obstacles to its prevalence. These are high initial equipment costs [25, 26], a required learning curve of the surgical staff [27, 28], and the current lack of multicenter, large-sample, long-term follow-up studies to provide the highest level of evidence [29, 30]. Specifically, compared with traditional techniques, the additional time required for system installation, registration, and instrument calibration in robot-assisted systems prolonged the duration of the operation and anesthesia. However, in this case series, we implemented several strategies to promote surgical safety and mitigate these risks. Preoperative simulation training and the progressively increasing proficiency of the surgical team contributed to enhanced intraoperative efficiency. Moreover, through rigorous multidisciplinary assessment and comprehensive preoperative evaluation, we sought to mitigate the physiological impact associated with the extended operative time. The postoperative recovery for patients in this cohort was uneventful, with no observed instances of vascular pedicle compromise (such as thrombosis or kinking) or other major complications.

We acknowledge several limitations of this preliminary study. Firstly, the retrospective design, small sample size (*n* = 5), and lack of a concurrent control group limit the generalizability of our findings and preclude definitive claims of clinical superiority. Consequently, comparisons with historical literature should be interpreted solely as contextual references due to inherent methodological heterogeneity across studies. Secondly, a 6-month follow-up limits the thorough evaluation of long-term biological outcomes, such as marginal bone loss or late implant stability, rendering our results primarily as a preliminary technical report. Finally, high equipment costs and the required surgical learning curve currently restrict widespread accessibility. Future multicenter, prospective randomized controlled trials with extended follow-ups are needed to evaluate the long-term clinical outcomes and potential role of robotic-assisted JIAD.

## Conclusion

The robot-assisted JIAD technique, based on the sandwich planning approach, appears to be a feasible approach that could enhance implant placement accuracy in complex mandibular reconstructions. However, further investigation through multicenter, long-term prospective studies is needed to evaluate the clinical applicability and potential role of this technology.

## Supplementary Information

Supplementary Material 1.

## Data Availability

No datasets were generated or analysed during the current study.

## Abbreviations

JIAD: Jaw in a day
ORN: Osteoradionecrosis
MRONJ: Medication-related osteonecrosis of the jaw
RAS: Robot-assisted surgery
VSP: Virtual surgical planning
CAD: Computer-aided design
CAM: Computer-aided manufacturing
3D: Three dimensions
CT: Computed tomography
CBCT: Cone-beam CT
ICC: Intraclass Correlation Coefficient
